# 5-*O*-Acetyl-Renieramycin T from Blue Sponge *Xestospongia* sp. Induces Lung Cancer Stem Cell Apoptosis

**DOI:** 10.3390/md17020109

**Published:** 2019-02-11

**Authors:** Wipa Chantarawong, Supakarn Chamni, Khanit Suwanborirux, Naoki Saito, Pithi Chanvorachote

**Affiliations:** 1 Cell-based Drug and Health Products Development Research Unit, Chulalongkorn University, Bangkok 10330, Thailand; chantarawong.wp@gmail.com; 2 Department of Pharmacology and Physiology, Faculty of Pharmaceutical Sciences, Chulalongkorn University, Bangkok 10330, Thailand; 3 Department of Pharmacognosy and Pharmaceutical Botany, Faculty of Pharmaceutical Sciences, Chulalongkorn University, Bangkok 10330, Thailand; supakarn.c@pharm.chula.ac.th (S.C.); Khanit.S@chula.ac.th (K.S.); 4 Graduate School of Pharmaceutical Sciences, Meiji Pharmaceutical University, 2-522-1 Noshio, Kiyose, Tokyo 204-8588, Japan; naoki@my-pharm.ac.jp

**Keywords:** 5-*O*-acetyl-renieramycin T, *Xestospongia* sp., cisplatin, lung cancer, anti-cancer, apoptosis

## Abstract

Lung cancer is one of the most significant cancers as it accounts for almost 1 in 5 cancer deaths worldwide, with an increasing incident rate. Management of the cancer has been shown to frequently fail due to the ability of the cancer cells to resist therapy as well as metastasis. Recent evidence has suggested that the poor response to the current treatment drugs and the ability to undergo metastasis are driven by cancer stem cells (CSCs) within the tumor. The discovery of novel compounds able to suppress CSCs and sensitize the chemotherapeutic response could be beneficial to the improvement of clinical outcomes. Herein, we report for the first time that 5-*O*-acetyl-renieramycin T isolated from the blue sponge *Xestospongia* sp. mediated lung cancer cell death via the induction of p53-dependent apoptosis. Importantly, 5-*O*-acetyl-renieramycin T induced the death of CSCs as represented by the CSC markers CD44 and CD133, while the stem cell transcription factor Nanog was also found to be dramatically decreased in 5-*O*-acetyl-renieramycin T-treated cells. We also found that such a CSC suppression was due to the ability of the compound to deplete the protein kinase B (AKT) signal. Furthermore, 5-*O*-acetyl-renieramycin T was able to significantly sensitize cisplatin-mediated apoptosis in the lung cancer cells. Together, the present research findings indicate that this promising compound from the marine sponge is a potential candidate for anti-cancer approaches.

## 1. Introduction

The oceans have long been recognized as a potential source of marine organisms that may be capable of producing structurally unique and biologically active compounds. Due to the enormously diversified environments found in the oceans, marine organisms are indeed founts of novel candidates for drug discovery [1]. Approximately 63% of new drugs currently being developed are related to natural products, including natural product-derived drugs, chemically-modified natural products, and synthetic compounds with a natural product as a pharmacophore. Over the period 1980–2008, about 60% of anti-cancer drugs were developed significantly from natural sources [2]. New concepts of cancer cell biology as well as cancer drug discovery are focused on a defined cancer type’s specific molecular targets. Cancer stem cells (CSCs) are a specialized rare population of cells within tumors that possess self-renewal, differentiation, and tumor forming abilities [3]. CSCs have also been shown to be a seed of cancer and a potentiating factor in cancer progression [4]. Emerging evidence has confirmed the strong relevance of CSCs and their impact on clinical outcomes, as CSCs have been shown to be resistant to therapeutic drugs and are the cause of metastasis; for instance, one study reported that CSCs are responsible for cisplatin resistance in lung cancer [5]. Besides, in vitro and in vivo studies have shown that cisplatin treatment can enrich CSCs in non-small-cell lung carcinoma (NSCLC) [6,7,8]. In lung cancer, CSCs can be characterized by an increase in stem cell transcription factors and cellular surface markers, such as CD44 and CD133 [5,9]. CD133 (Prominin 1) is a cell surface glycoprotein that has been identified as an important molecular marker of stem-like cells. Recent research showed that CD133 expression is related to the levels of resistance-mediated proteins in patients with NSCLCs [10]. CD133^+^ cancer cells exhibit significant resistance to anti-tumor treatment, including chemotherapy [10]. A recent study indicated that cisplatin could increase the ratio of CD133^+^ cells in lung cancer [11]. Accumulating data point out the important role of the AKT signaling pathway in the tumorigenicity of CSCs [12]. It has been reported that AKT inhibitors could suppress the colony formation of CSCs, which suggests they might be potential agents for suppressing CSCs in cancer chemotherapy [13].

**Figure 1:**
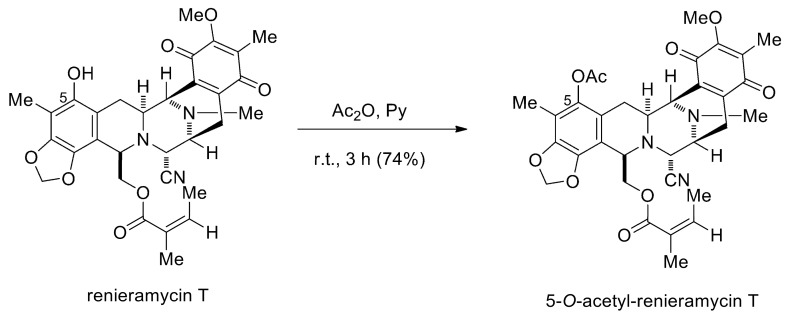
The synthesis of 5-*O*-acetyl-renieramycin T.

Renieramycins A−Y are a series of tetrahydroisoquinoline marine alkaloids isolated from *Xestospongia* sp., which is a marine blue sponge found in the seas around Thailand and the Philippines [14,15,16,17,18,19]. These renieramycin derivatives contain the chemical structures and biological activities related to other isoquinoline natural products, such as naphthyridinomycins, quinocarcins, saframycins, and ecteinascidins [14], which exhibit diverse bioactivities, such as antitumor, antibacterial, antiviral, anticoagulant, anti-inflammatory, anti-Alzheimer, and anticonvulsant activities [20]. Among the renieramycins family, renieramycin T, a renieramycin–ecteinascidin hybrid marine natural product, has recently become an interesting target for synthetic and biological studies regarding a highly substituted phenol and a condensed 1,3-dioxole ring, which are similar to the left-hand-side carbon framework of those in ecteinascidins [21,22]. The addition of an acetyl group by esterification of the phenol moiety of renieramycin T furnishes 5-*O*-acetyl renieramycin T (*O*-acetyl RT) (Figure 1), having an identical left-hand-sided structure to the U.S. Food and Drug Administration (FDA)-approved anti-cancer drug Ecteinascidin 743 (Yondelis^®^) [23]. It has been reported that *O*-acetyl RT and other renieramycins, such as renieramycin M and renieramycin T, have a strong cytotoxicity, with inhibitory concentration (IC_50_) values in the range of nanomolar concentrations against many human cancer cell lines, including colon cancer HCT116 and DLD1 cells, breast cancer MDA-MB-435 cells, pancreatic cancer AsPC1 cells, lung carcinoma QC56 cells, and non-small-cell lung cancer cells [19,24,25]. However, the effect of *O*-acetyl RT and its mechanism on human NSCLC has not yet been elucidated. Herein, we report that *O*-acetyl RT synthesized from renieramycin T induced apoptosis, suppressed CSCs, and sensitized NSCLC H292 cells to cisplatin. These findings might benefit the development and further investigation of this compound as a sensitizer of NSCLC to cisplatin.

## 2. Results

### 2.1. O-Acetyl RT Reduced the Cell Viability and Induced the Apoptosis of Non-Small-Cell Lung Carcinoma (NSCLC) Cells

**Figure 2:**
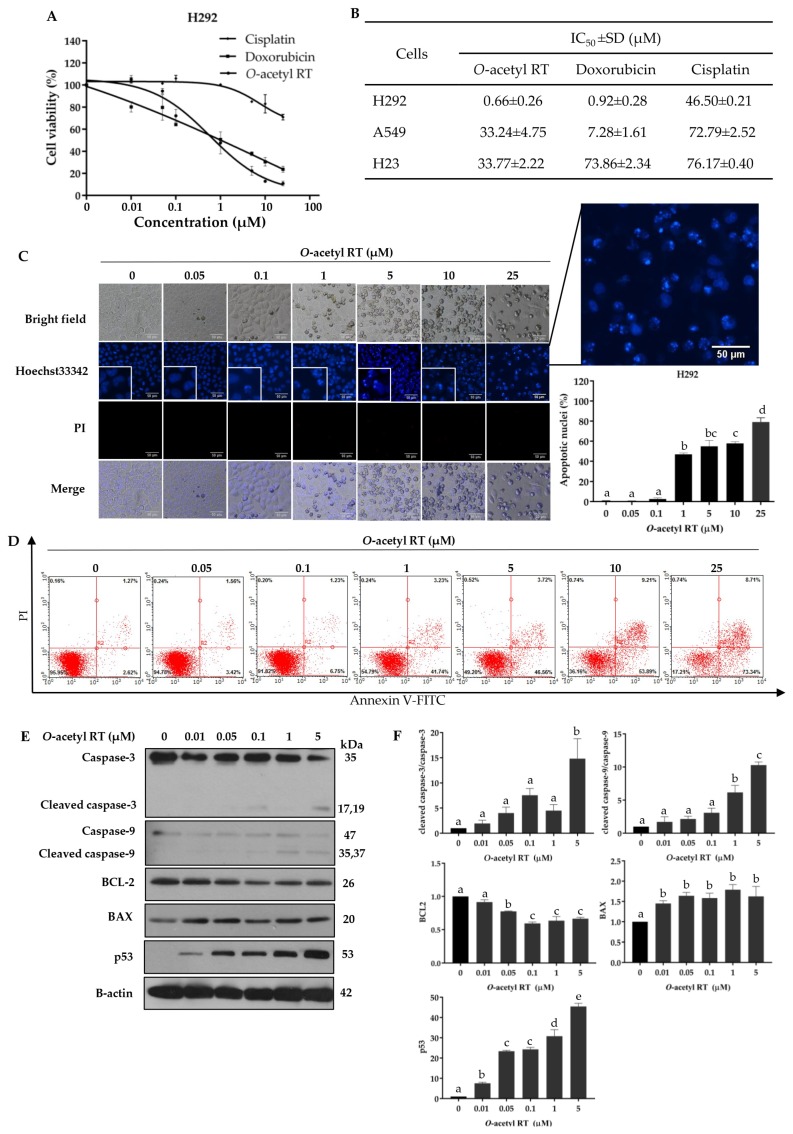
*O*-acetyl RT reduces cell viability and induces apoptosis in non-small-cell lung carcinoma (NSCLC) cells. (**A**) H292 cells were treated with various concentrations of *O*-acetyl RT, cisplatin, and doxorubicin (0–25 µM) for 24 h. Cell viability was determined by MTT assay. (**B**) The half-maximal inhibitory concentration (IC_50_) of each cell line at 24 h was calculated. (**C**) The nuclei of H292 cells treated with *O*-acetyl RT were stained with Hoechst 33342/propidium iodide (PI) and calculated as a percentage compared with nontreated control cells. (**D**) Apoptotic and necrotic cell death were determined using the Annexin V/PI staining assay. (**E**) The expression levels of apoptosis-associated proteins, caspase-3, cleaved caspase-3, caspase-9, cleaved caspase-9, BCL-2, BAX, and p53 proteins in H292 cells treated with *O*-acetyl RT (0–5 µM) for 24 h were examined by Western blot analysis. To confirm equal loading of the protein samples, the blots were reprobed with the β-actin antibody. (**F**) Relative protein levels were quantified by densitometry. Data represent the mean ± standard deviation (SD) (*n* = 3). Bars labeled with different letters (a, b, c, d, e) are significantly different at *p* < 0.05.

Before the sensitizing effect of *O*-acetyl RT on cisplatin-mediated cancer cell death was determined, we investigated the cytotoxic profile of *O*-acetyl RT compared with doxorubicin and cisplatin, the standard chemotherapeutic drugs for lung cancer therapy, using the MTT cell viability assay. Human lung cancer H292 cells were incubated with various concentrations (0–25 µM) of compounds for 24 h. The results showed that *O*-acetyl RT had a greater cytotoxic effect on H292 cells than that of doxorubicin and cisplatin (Figure 2A,B). *O*-acetyl RT was considered non-toxic at concentrations ≤ 0.05 µM for H292 cells. Moreover, the cytotoxic effect of *O*-acetyl RT was also assessed in A549 and H23 cells. The results showed that *O*-acetyl RT exhibited cancer-suppressing activity in a dose-dependent manner in all three of the NSCLC cell lines but was more effective in H292 cells (Figure 2B).

To determine whether the anti-cancer effect of *O*-acetyl RT was related to apoptosis, we treated H292 cells with *O*-acetyl RT (0–25 µM) for 24 h and then quantified the apoptotic cells using the Hoechst 33342/propidium iodide (PI) double staining assay. We found that *O*-acetyl RT was able to induce apoptosis of H292 cells in a dose-dependent manner (Figure 2C). The result revealed the presence of apoptotic nuclei with condensed or fragmented nuclei in the cells treated with *O*-acetyl RT at concentrations ≥ 1 μM, whereas the number of apoptotic cells was not changed in cells treated with lower concentrations (< 1 μM) (*F*_6,14_ = 386.32, *p* < 0.0001). Moreover, necrosis cell death was not detected under all treatments. To confirm the apoptosis-inducing effect of *O*-acetyl RT, apoptotic cells were determined using the Annexin V/PI apoptosis assay. *O*-acetyl RT caused an increase in the number of early-apoptotic cells dependent on the treated concentrations (Figure 2D). As shown in Figure 2D, the percentage of early-apoptotic cells was 41.76%, 46.56%, 53.89%, and 73.34% in the H292 cells treated with *O*-acetyl RT at concentrations of 1, 5, 10, and 25 µM, respectively. Together, these data indicate that *O*-acetyl RT decreased cell viability and increased apoptotic cells in H292 cells.

### 2.2. O-Acetyl RT Induced Apoptosis through p53 Activation

Having shown the potential of apoptosis induction, we then confirmed the apoptosis effect of *O*-acetyl RT using specific apoptotic markers, including the activation of caspase-3 and caspase-9. The cells were treated with *O*-acetyl RT (0–5 µM). The key proteins controlling apoptosis were determined by Western blot analysis. As shown in Figure 2E,F, 5 μM of *O*-acetyl RT significantly induced the activation of caspase-3 compared to the non-treated cells (*F*_5,12_ = 14.63, *p* = 0.0026). In agreement with such results, the expression of the active form of caspase-9 was found to be significantly upregulated in H292 cells treated with *O*-acetyl RT at 1 and 5 µM (*F*_5,12_ = 57.63, *p* < 0.0001).

We further evaluated the underlying mechanism of apoptosis induction by investigating the major regulators of p53-dependent apoptosis, such as BCL-2, BAX, and p53, which is one of the important mechanisms of anti-cancer drug action [26,27,28]. Furthermore, the BCL2 family proteins are important mediators for chemotherapeutic resistance [29,30]. Western blot analysis showed that there was an increase in the expression of BAX (*F*_5,12_ = 9.021, *p* = 0.0093) and p53 (*F*_5,12_ = 219, *p* < 0.0001), and a decrease in the expression of BCL-2 (*F*_5,12_ = 51.31, *p* < 0.0001) in *O*-acetyl RT-treated H292 cells compared to non-treated cells. (Figure 2E,F). Taken together, these results indicated that *O*-acetyl RT induces apoptosis via activating p53 as well as by suppressing the anti-apoptotic BCL2 protein and inducing the pro-apoptotic BAX protein in H292 cells.

### 2.3. O-Acetyl RT Suppresses Cancer Stem Cell Signals in H292 Cells

**Figure 3:**
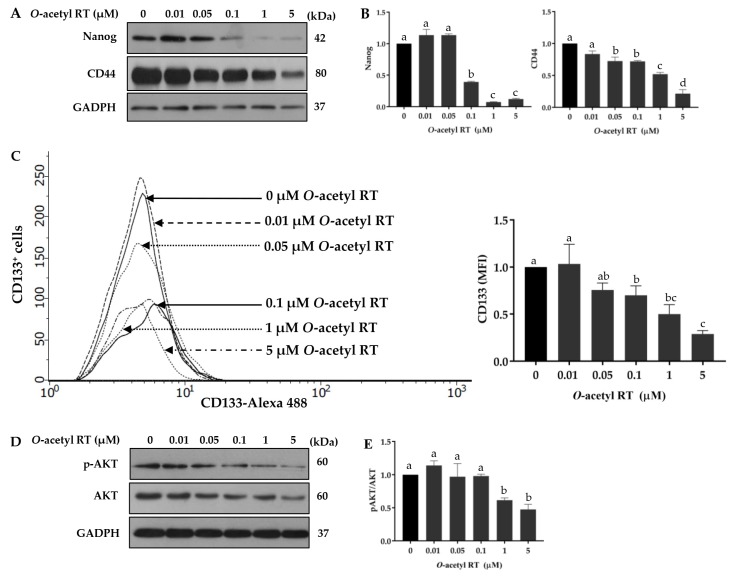
*O*-acetyl RT inhibits cancer stem cell signals. Cells were treated with *O*-acetyl RT (0–5 µM) for 24 h. The expression levels of cancer stem cell markers CD44 and Nanog in H292 cells were determined by Western blot analysis. (**A**) The blots were reprobed with the GADPH antibody. (**B**) Relative protein levels were determined by densitometry. (**C**) The CD133^+^ cells were determined by flow cytometry. (**D**) Cellular levels of activated AKT (p-AKT) and total AKT were examined by Western blot analysis. (**E**) The immunoblot signals were quantified by densitometry. Data represent the mean ± SD (*n* = 3). Bars labeled with different letters (a, b, c, d) are significantly different at *p* < 0.05.

CSCs are known to be a driver of aggressive behaviors and poor prognosis in various cancers [22]. For lung cancer, the AKT pathway and its downstream signals have been frequently shown to contribute CSC phenotypes [12]. Therefore, we determined whether *O*-acetyl RT was able to suppress such CSC signals. Cells were treated or left untreated with the compound for 24 h and the levels of lung CSC marker CD44 and pluripotent transcription factor Nanog were determined by Western blot analysis. As shown in Figure 3A,B, *O*-acetyl RT at the concentrations ≥ 0.1 and ≥ 0.05 significantly decreased the cellular levels of Nanog (*F*_5,12_ = 333.8, *p* < 0.0001) and CD44 (*F*_5,12_ = 77.13, *p* < 0.0001), respectively. In addition, this CSC-suppressing activity of the compound was supported by the depletion of CD133-positive (CD133^+^) cells (*F*_5,12_ = 21.14, *p* < 0.0001) in the *O*-acetyl RT-treated cells, as determined by flow cytometry (Figure 3C). The corresponding upstream signals, including AKT, were monitored by Western blotting. We found that *O*-acetyl RT caused a reduction in active AKT (*F*_5,12_ = 15.27, *p* = 0.0023) in H292 cells (Figure 3D,E).

### 2.4. O-Acetyl RT Increases Sensitivity of H292 Cells to Cisplatin

**Figure 4:**
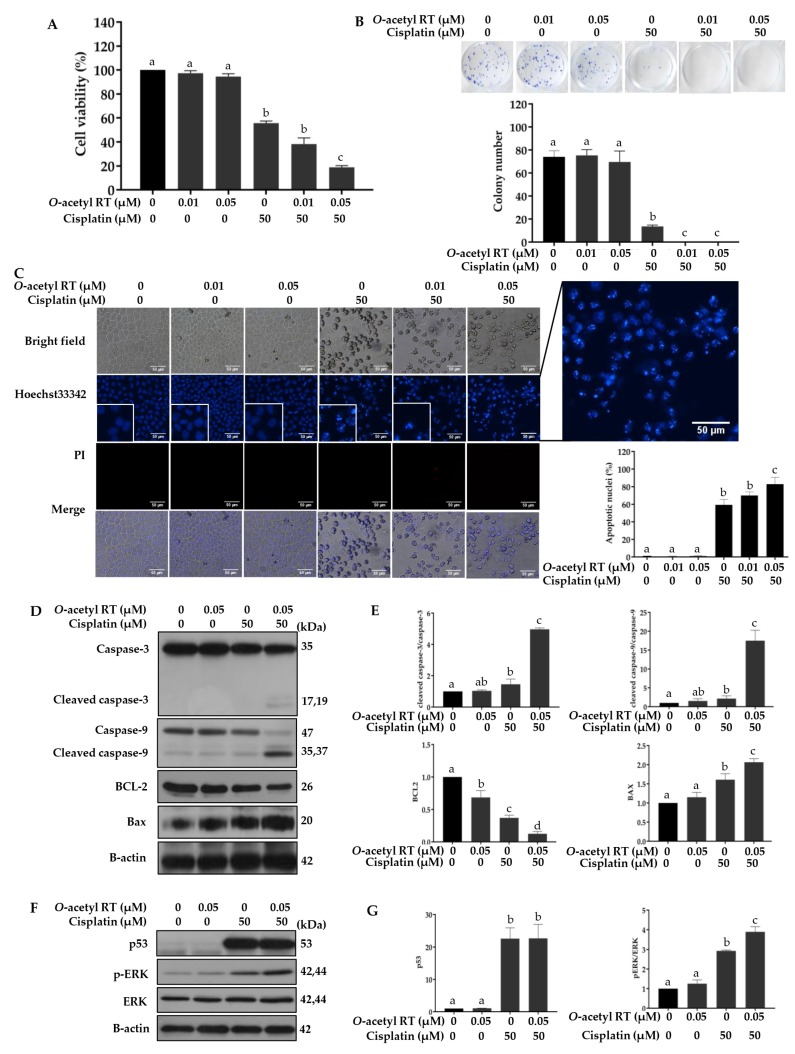
*O*-acetyl RT sensitizes H292 to cisplatin. (**A**) Cell viability of NSCLC H292 cells treated with 0.01 and 0.05 µM of *O*-acetyl RT for 24 h prior to treatment with 50 µM of cisplatin was determined using the MTT assay. (**B**) Effect of *O*-acetyl RT sensitization on cell growth of H292 cells was observed using a colony formation assay. (**C**) Hoechst 33342/PI double staining was used to determine cell apoptosis. (**D**) The expression levels of apoptosis-associated proteins in H292 cells treated with 0.05 µM of *O*-acetyl RT for 24 h prior to treatment with 50 µM of cisplatin were assessed by Western blotting, and (**E**) the relative expression was calculated using densitometry. (**F**) The expression levels of activated ERK (p-ERK), total ERK, and p53 were also examined using Western blotting, and (**G**) the relative expression was calculated using densitometry. Data represent the mean ± SD (*n* = 3). Bars labeled with different letters (a, b, c, d) are significantly different at *p* < 0.05.

To test whether *O*-acetyl RT could sensitize H292 cells to cisplatin, the cells were pretreated with non-toxic concentrations (0.01 or 0.05 µM) of *O*-acetyl RT for 24 h, followed by treatment with 50 µM cisplatin for 24 h, and cell viability was measured using MTT assay. Figure 4A shows that the H292 cells were robustly sensitized to cisplatin by pretreatment with 0.05 μM *O*-acetyl RT (Figure 4A) (*F*_5,12_ = 698.3, *p* < 0.0001). The cell viability of H292 cells treated with cisplatin was reduced by 56%, whereas a combination of 0.01 or 0.05 µM *O*-acetyl RT with cisplatin was more effective than cisplatin alone and reduced cell viability by 62% and 81%, respectively. This effect was further confirmed using a colony formation assay (Figure 4B) (*F*_5,12_ = 178, *p* < 0.0001). Figure 4C also shows that the combination treatments of 0.05 µM *O*-acetyl RT and 50 µM cisplatin significantly induced apoptosis compared to the group treated with cisplatin alone (*F*_5,12_ = 238.3, *p* < 0.0001). Western blot analysis showed that the pretreatment of 0.05 µM *O*-acetyl RT significantly increased the expression of the active form of caspase-3 (*F*_3,8_ = 237.9, *p* < 0.0001) and caspase-9 (*F*_3,8_ = 60.9, *p* = 0.0009) (Figure 4D,E). Figure 4D,E also show that the level of the anti-apoptotic protein BCL-2 was significantly downregulated (*F*_3,8_ = 80.87, *p* = 0.0005) and pro-apoptotic protein BAX was significantly upregulated (*F*_3,8_ = 38, *p* = 0.0021) in the *O*-acetyl RT pretreatment. Pretreatment with 0.05 µM *O*-acetyl RT could increase the expression of p53 (*F*_3,8_ = 41.83, *p* = 0.0018) but the level of p53 expression was not altered in the *O*-acetyl RT pretreatment group when compared to the group treated with cisplatin alone (*p* > 0.9999). It has been reported that cisplatin can activate the ERK pathway leading to the induction of p53-dependent apoptosis in lung cancer cells [31]. In line with this, the results from Figure 4F,G show that pretreatment with *O*-acetyl RT clearly increased the p-ERK level in H292 cells (*F*_3,8_ = 142.9, *p* < 0.0002).

### 2.5. O-Acetyl RT Reduces Cisplatin-Induced CD133^+^ Cells Subpopulation in H292 Cells

**Figure 5:**
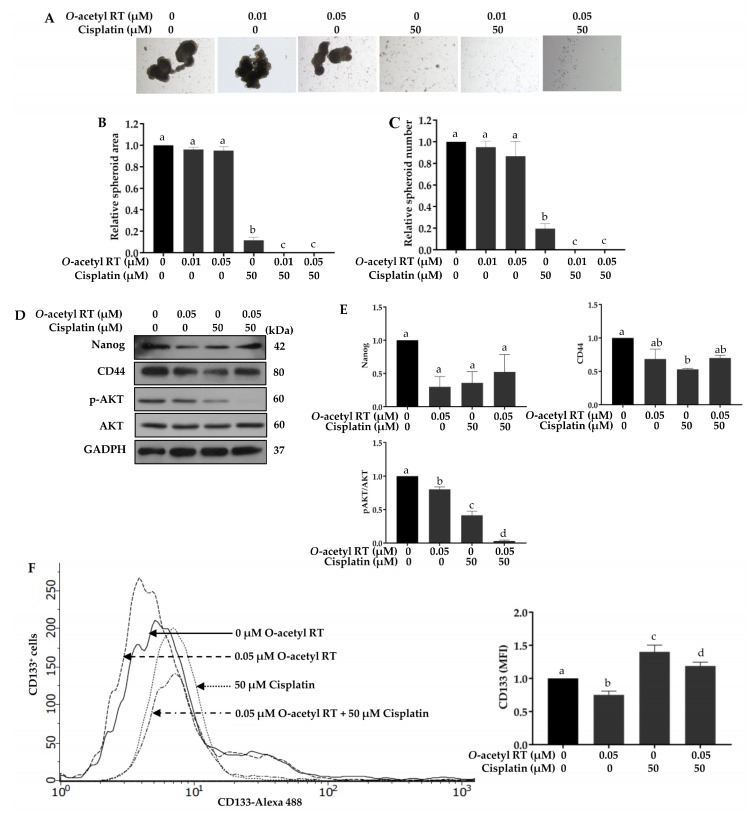
Pretreatment with *O*-acetyl RT reduces the cisplatin-induced enrichment of CD133^+^ cells. After treatment with 0.01 and 0.05 µM of *O*-acetyl RT for 24 h, following treatment with 50 µM cisplatin, H292 cells were suspended and subjected to spheroid formation assay. (**A**) Spheroids at day 7 were determined by phase-contrast microscopy, and (**B**,**C**) the spheroid number and area were quantified. (**D**) The expression levels of Nanog, CD44, p-AKT, total AKT, and p53 in H292 cells treated with 0.05 µM of *O*-acetyl RT for 24 h prior to treatment with 50 µM of cisplatin were determined using Western blotting, and (**E**) the relative expression was assessed using densitometry. (**F**) The CD133^+^ cells were measured by flow cytometry. Data represent the mean ± SD (*n* = 3). Bars labeled with different letters (a, b, c, d) are significantly different at *p* < 0.05.

We further observed the inhibition of cisplatin-induced stemness with *O*-acetyl RT sensitization. The spheroid number and area of the group pretreated with *O*-acetyl RT, following by treatment with 50 µM cisplatin, was also lower than those of the group treated with cisplatin alone (Figure 5A–C). We also observed the expression of stemness-related proteins using Western blot analysis. Pretreatment with 0.05 µM *O*-acetyl RT could reduce the expression of the Nanog (*F*_3,8_ = 6.624, *p* = 0.0496) and CD44 (*F*_3,8_ = 12.84, *p* = 0.0161) but the results were not significantly different from those of the group pretreated with *O*-acetyl RT and the group treated with cisplatin alone (Figure 5D,E). In addition, pretreatment with *O*-acetyl RT could also significantly decrease the phosphorylation of AKT (*F*_3,8_ = 269.3, *p* < 0.0001), a critical gene related to survival and stemness, whereas total AKT was not changed in comparison to that of cisplatin used alone. Previous results have suggested that cisplatin increased CD133^+^ cells subpopulation in NSCLC cells, which is a responsible for drug resistance [12]. Thus, we observed the effect of *O*-acetyl RT on the number of CD133^+^ cells subpopulation in NSCLC using flow cytometry. As shown in Figure 5F, the CD133^+^ cells subpopulation was increased in H292 cells treated with cisplatin alone, while pretreatment with *O*-acetyl RT was able to reduce the number of CD133^+^ cells subpopulation (*F*_3,8_ = 56.3, *p* < 0.0001). These results indicate that pretreatment with *O*-acetyl RT significantly reverses cisplatin-induced CD133^+^ cells subpopulation in H292 cells.

## 3. Discussion

5-*O*-acetyl-renieramycin T is a derivative of renieramycin T, which was isolated from the blue sponge *Xestospongia* sp. found in the seas around Thailand [24] and the Philippines [15,19]. A previous study reported that this synthetic derivative possesses cytotoxicity to many human cancer cell lines, including human colon carcinoma (HCT116), human ductal breast epithelial tumor (T47D), human pancreatic adenocarcinoma (AsPC1), and human lung carcinoma (QG56), for which its IC_50_ at 96 h ranges from 0.01 to 0.12 µM [14]. In this study, we determined the cytotoxicity effect of *O*-acetyl RT on NSCLC cells, H292, A549, and H23 cells. H292 cells were more sensitive to *O*-acetyl RT than A549 and H23 cells. Our data indicated that *O*-acetyl-RT has a strong cytotoxic effect on human H292 cell, with an IC_50_ at 24 h of 0.66 µM. Moreover, *O*-acetyl RT can also induce p53-dependent apoptosis in H292 cells (Figure 2.). The tumor suppressor p53 is a transcription factor that regulates numerous genes associated with cell cycle arrest, DNA repair, and apoptosis [32]. p53 is activated by various stress stimuli, such as irradiation and DNA damage [33]. The exposure of H292 cells to *O*-acetyl RT (0–5 µM) for 24 h caused a dose-dependent increase in p53 protein level. *O*-acetyl RT at concentrations ≥ 0.1 µM was sufficient to activate the induction of p53 and apoptosis in H292 cells. Treatment with *O*-acetyl RT at 0.05 µM for 24 h increased p53 protein levels but did not induce apoptosis in H292 cells (Figure 2). Moreover, the expression levels of p53 induced by this concentration were decreased to basal levels after incubation with the medium without cisplatin for 24 h (Figure 4F,G).

Cisplatin has been widely used as a chemotherapeutic drug to treat various types of cancer, including NSCLC [3,34]. However, its anti-cancer efficacy is limited due to its side effects and resistance [35]. Cisplatin resistance is contributed by various mechanisms, including reduced intracellular cisplatin accumulation, increased DNA damage repair, increased anti-apoptotic BCL2 levels, and an inactivated apoptotic-related pathway [36]. Accumulating data has indicated that cisplatin induces apoptosis via causing DNA damage, which triggers activation of the p53 protein [37,38,39]. Herein, cisplatin induced p53-dependent apoptosis and, of note, its efficiency was increased by *O*-acetyl RT. However, the expression levels of p53 were not significantly different between treatment with cisplatin alone and pre-treatment with *O*-acetyl RT at 0.05 µM. This result suggests that *O*-acetyl RT sensitized H292 cells to cisplatin-induced apoptosis in a p53-independent manner. In addition to p53, it has been reported that the ERK signaling pathway can be activated by cisplatin and is a key pathway of p53-dependent apoptosis induced by cisplatin in many cancer cells, including lung cancer cells [29,40,41]. Nevertheless, the ERK pathway is activated in various types of cancers, which activates cancer growth, angiogenesis, and invasion, and suppresses apoptosis [42]. The present study showed that *O*-acetyl RT increased cisplatin-induced ERK activation. Thus, *O*-acetyl RT sensitized H292 cells to cisplatin through inducing p53-mediated apoptosis via activating the ERK pathway (Figure 4).

The BCL-2 family of proteins, including anti-apoptotic BCL-2 and pro-apoptotic BAX, are critical regulators of the mitochondrial apoptotic pathway and they have been associated with a more aggressive treatment and drug resistance in cancer chemotherapy [43,44,45]. In addition, BCL-2 has been reported as a target gene for many signaling pathways, including the ERK pathway [46,47,48]. The present results indicated that the treatment of cisplatin increased the phosphorylation of ERK proteins, which downregulated the downstream anti-apoptotic BCL-2 protein and upregulated the pro-apoptotic BAX protein in H292 cells. With a change of balance of BCL-2 and BAX, cisplatin increased apoptosis in H292 cells and *O*-acetyl RT could promote its efficiency.

Previous studies have reported that CSCs drive cancer dissemination and relapse [4,49]. Herein, our results indicated that *O*-acetyl-RT suppressed CSCs by the reduction of Nanog and CD44 expression and the number of CD133^+^ cells via an AKT-dependent pathway (Figure 3). Nanog is a transcription factor that is responsible for the maintenance of self-renewal and pluripotency in cancer stem cells under regulation by the AKT pathway [50,51]. It has been reported that a high expression of Nanog can induce CSC-like phenotypes in lung cancer [52]. CD44 and CD133 have been reported as cancer stem cell markers in lung cancer [53,54,55].

Proliferation and maintenance of CSCs is regulated by other signaling pathways including Wnt, Notch, Hedgehog, TGFβ, STAT3 and PI3K/AKT [56,57]. Many studies have shown that AKT is a critical signal that promotes drug resistance and maintains cancer stem-like cell in various cancers including lung cancer [50,58]. Activation of AKT can occur through phosphatidylinositol 3-kinases (PI3Ks) that regulate many cellular processes, including cell growth and survival, via receptor tyrosine kinases [59]. Moreover, it has been demonstrated that the inhibition of Akt pathway could suppress the expression of CD133 and CD44 [12,13]. CD133 and CD44 often serve as important stemness markers in NSCLC [53,60]. A recent study indicated that the pluripotency genes (OCT4, SOX2, and NANOG) were expressed in CD44+ cells which are more resistant to cisplatin treatment than CD44− cells [54]. CSCs are also believed to play a role in chemotherapeutic resistance [8]. Growing evidence suggests that cisplatin treatment could induce the expression of CSC markers in NSCLC cells [6,61]. It has been reported that cisplatin-resistant CD133^+^ cells with a self-renewal property, chemoresistance, and a high tumorigenic capability were observed in NSCLC cell lines and in in vivo xenograft models [62]. The inhibition of CD133 expression diminished stemness properties, increased apoptosis via the regulation of BCL-2 and BAX, and increased chemoradiosensitivity in liver cancer stem cells [63]. Interestingly, our data indicated that *O*-acetyl RT could reduce the cisplatin-induced CD133^+^ cell subpopulation in H292 cells (Figure 5).

## 4. Materials and Methods

### 4.1. Reagents and Antibodies

Dulbecco’s Modified Eagle’s Medium (DMEM) medium, Roswell Park Memorial Institute (RPMI) 1640 medium, fetal bovine serum (FBS), penicillin/streptomycin, L-glutamine, phosphate-buffered saline (PBS) and trypsin-EDTA were obtained from Gibco (Grand Island, NY, USA). 3-(4,5-dimethylthiazol-2-yl)-2,5-diphenyltetrazoliumbromide (MTT), dimethyl sulfoxide (DMSO), Hoechst33342, propidium iodide (PI), cisplatin, doxorubicin and bovine serum albumin (BSA) were obtained from Sigma-Aldrich, Co. (St. Louis, MO, USA). The following primary antibodies, caspase-9 (#9502), caspase-3 (#9662), p53 (#9282), BCL-2 (#4223), BAX (#5023), AKT (#9272), phosphorylated AKT (#4060), ERK (#4695), phosphorylated ERK (#4370), Nanog (#4903), CD44 (#9502), GADPH (#5174) and β-actin (#4970), were obtained from Cell Signaling Technology (Danvers, MA, USA). CD133 (#CA1217) was obtained from Cell Applications (San Diego, CA, USA). The respective secondary antibodies, anti-rabbit IgG (#7074) and anti-mouse (#7076), were obtained from Cell Signaling Technology (Danvers, MA, USA).

### 4.2. Preparation of O-Acetyl RT

The Thai blue sponge *Xestospongia* sp. was collected by scuba diving at a depth of 3–5 m in the vicinity of Sichang Island, Chonburi Province, Thailand, in October 2016 with the assistance of the Aquatic Resources Research Institute, Chulalongkorn University. The fresh sponge was identified based on the light bluish-gray color, bulbous surface lobes, numerous and moderate size of oscules and easily crumble texture [16]. The collected sponge was kept frozen at –20 °C until used.

The collected blue sponge was homogenized and suspended in the phosphate buffer solution (pH 7). Then, a solution of 10% potassium cyanide (KCN) in the phosphate buffer solution (pH 7) was added dropwise to the suspension until the overall KCN concentration reached 10 mM [16]. Thereafter, the mixture was macerated for 48 h with methanol. The extract was filtered and the filtrate was concentrated under reduced pressure to obtain the aqueous methanolic solution. The combined aqueous methanolic solution was partitioned with hexane and ethyl acetate, consecutively. Then, the volatile solvent is removed to give a crude residue. The residue was subjected to silica gel chromatography using the gradient solvent mixture of hexane, ethyl acetate, and methanol to furnish renieramycin T as amorphous powder.

The naturally produced renieramycin T was employed as a synthetic precursor for chemical modification to yield *O*-acetyl RT by esterification. Renieramycin T (10.2 mg, 0.018 mmol) was dissolved in dry pyridine (1.0 mL). Acetic anhydride (2.5 μL, 0.027 mmol) was added to the reaction mixture, which was then stirred at room temperature (25 °C) under an argon atmosphere for 3 h (Figure 1). Then, the reaction was quenched by the addition of water (5 mL) and extracted with CH_2_Cl_2_ (10 mL, 3 times). The organic layers were combined, washed with brine (30 mL), dried over anhydrous Na_2_SO_4_, filtered and concentrated under reduced pressure to obtain a residue. The crude product was purified by flash chromatography using silica gel and a mixture of hexanes:EtOAc (4:1) solution as stationary phase and mobile phase, respectively to furnish *O*-acetyl RT (8.1 mg, 74%) as a yellow amorphous solid. The spectroscopic data of *O*-acetyl RT was matched with the previous report [23].

### 4.3. Preparation of O-Acetyl RT and Cisplatin Stock Solution

*O*-acetyl RT was prepared in DMSO and was further diluted in a complete medium to the desired concentrations. The final concentration of DMSO in each treatment was less than 0.1% which showed no signs of cytotoxicity. Cisplatin was dissolved in 0.9% sodium chloride (NaCl) and was diluted in complete medium to provide the indicated working concentrations. The solution was aliquoted and stored at −20 °C until further use.

### 4.4. Cell Lines and Culture

Human non-small cell lung cancer (NSCLC) cell lines, H292, A549 and H23 cells were obtained from the American Type Culture Collection (Manassas, VA, USA). H292 and H23 cells were cultured in RPMI 1640 medium. A549 cells were cultured in DMEM medium. The medium was supplemented with 10% FBS, 2 mM L-glutamine and 100 units/ml of each penicillin and streptomycin at 37 °C with 5% CO_2_ in a humidified incubator.

### 4.5. Cell Viability Assay

Cell viability inhibition effect of *O*-acetyl RT on NSCLC cells was determined by MTT assay. In brief, cells were seeded into 96-well plate at 1 × 10^4^ cells/well, allowed to adhere overnight and then treated with different concentration of *O*-acetyl RT (0, 0.01, 0.05, 0.1, 1, 5, 10, 25 µM) for 24 h. After that, 100 μL of MTT solution (400 µg/ml) was added, incubated for another 4 h at 37 °C. The formazan crystal product was dissolved in 100 μL of DMSO. The optical density was measured at 570 nm using a microplate reader (Anthros, Durham, NC, USA). For *O*-acetyl RT sensitization in H292 cells, the cells were exposed to *O*-acetyl RT for 24 h, and then after washout of *O*-acetyl RT, cells were incubated in medium with or without cisplatin (50 µM) for an additional 24 h.

### 4.6. Colony Formation Assay

H292 cells pre-treated with *O*-acetyl RT at non-toxic concentration (0, 0.01 and 0.05 μM) for 24 h were seeded in 24 well plates at a density of 100 cells/well and incubated for 7 days. The colony was fixed with methanol at 4 °C for 15 min and stained with 0.1% crystal violet for 15 min. Colony formation was assessed using a phase-contrast microscope (Olympus IX5, Tokyo, Japan) equipped with a DP70 digital camera system (Olympus, Tokyo, Japan).

### 4.7. Spheroid Formation Assay

H292 cells pretreated with 0.01 or 0.05 µM *O*-acetyl RT, followed by incubation in medium with or without 50 µM cisplatin, were cultured in serum-free medium in 24-well ultralow attachment plates at a cell density of 2500 cells/well. After 7 days, the spheroids were determined using phase-contrast microscopy. The relative spheroid number and area were calculated compared with those of the non-treated cells.

### 4.8. Nuclear Staining Assay

Apoptotic cell death was determined by nuclear staining with a Hoechst 33342 and PI. After being treated with the indicated concentration, H292 cells were stained with 10 μg/mL Hoechst 33342 for 15 min at 37 °C and then stained with 5 μg/mL PI. Cells were imaged randomly using the fluorescent microscope (Olympus IX5, Tokyo, Japan). Apoptotic cells with nuclear condensation and DNA fragmentation were analyzed and expressed as a percentage of apoptotic cells.

### 4.9. Annexin V and Propidium Iodide Apoptosis Assay

After treatment with *O*-acetyl RT, H292 cells were collected and washed in cold PBS, pH 7.4. The cells were then dispersed in binding buffer containing annexin V-FITC and PI as recommended in the manufacturer’s instructions (ImmunoTools, Friesoythe, Germany). Live, apoptotic and necrotic cells were determined using Guava easyCyte flow cytometer (EMD Millipore, Hayward, CA, USA).

### 4.10. Western Blot Analysis

After *O*-acetyl RT treatment, H292 cells were incubated with RIPA lysis buffer supplemented with protease inhibitor cocktail (Roche diagnostics, Indianapolis, IN, USA) for 1 h on ice. The extracted proteins (50 μg) were separated by sodium dodecyl sulfate polyacrylamide gel electrophoresis (SDS-PAGE) and further transferred to 0.45 μm nitrocellulose membranes (Bio-Rad laboratories, Hercules, CA, USA). The membranes were blocked in Tris-buffer saline containing 0.1% Tween 20 and 5% non-fat dry milk for 1 h at room temperature and incubated with the appropriate primary antibodies at 4 °C overnight. The membranes were further incubated with horseradish peroxidase (HRP)-conjugated secondary antibodies for 2 h at room temperature. The membranes were detected using an enhancement chemiluminescent detection system (Supersignal West Pico, Pierce, Rockford, IL, USA) and subsequently exposed to X-ray film. The protein band was analyzed using ImageJ software (version 1.52, National Institutes of Health, Bethesda, MD, USA).

### 4.11. Flow Cytometry Analysis

To determine CD133 expression by flow cytometry, H292 cells treated with *O*-acetyl RT were trypsinized and incubated in PBS supplemented with 3% BSA for 30 min on ice. After that, cells were incubated with an anti-CD133 antibody for 1 h at 4 °C. The cells were then incubated with an Alexa488-conjugated secondary antibody (Catalog no. A11037, Invitrogen, Eugene, OR, USA) for 30 min at 4 °C in the dark and washed again before analysis using Guava easyCyte flow cytometer (EMD Millipore, Hayward, CA, USA).

### 4.12. Statistical Analysis

All data from at least three independent experiment are expressed as mean ± standard deviation (SD). Statistical differences between multiple groups were analyzed using analysis of variance (ANOVA), followed by Turkey’s post-hoc test for individual comparisons at the *p* < 0.05 significance level.

## 5. Conclusions

Our results provide novel and significant data indicating that *O*-acetyl RT is able to induce apoptosis and suppress the expression of CSC markers in H292 cells. We also found that *O*-acetyl RT had the ability to increase cisplatin-induced apoptosis and to decrease the number of cisplatin-induced CD133^+^ cells. These results provide novel and significant data indicating that *O*-acetyl RT might be a promising candidate as a sensitizer in cancer chemotherapy for reducing resistance and suppressing tumor progression.
